# Alcohol and Difficulty Conceiving in the SUN Cohort: A Nested Case-Control Study

**DOI:** 10.3390/nu7085278

**Published:** 2015-07-27

**Authors:** Cristina Lopez-del Burgo, Alfredo Gea, Jokin de Irala, Miguel A. Martínez-González, Jorge E. Chavarro, Estefania Toledo

**Affiliations:** 1 Department of Preventive Medicine and Public Health, School of Medicine, University of Navarra, 31008 Pamplona, Navarra, Spain; E-Mails: ageas@alumni.unav.es (A.G.); jdeirala@unav.es (J.I.); mamartinez@unav.es (M.A.M.-G.); etoledo@unav.es (E.T.); 2 Institute for Culture and Society, University of Navarra, 31008 Pamplona, Navarra, Spain; 3 CIBER Fisiopatología de la Obesidad y Nutrición, Instituto de Salud Carlos III, 28029 Madrid, Spain; 4 IdiSNA, Navarra Institute for Health Research, 31008 Pamplona, Navarra, Spain; 5 Department of Epidemiology, Harvard T. H. Chan School of Public Health, 02115 Boston, MA, USA; E-Mail: jchavarr@hsph.harvard.edu; 6 Department of Nutrition, Harvard T. H. Chan School of Public Health, 02115 Boston, MA, USA

**Keywords:** fertility, nested case-control study, diet, alcohol, wine, beer

## Abstract

The role of alcohol on fertility remains unclear. We aimed to investigate the association between alcohol and specific alcoholic beverages consumption and the risk of difficulty getting pregnant. We used a case-control study nested within the Seguimiento Universidad de Navarra (SUN) cohort, a prospective, dynamic and multipurpose cohort of 21,705 Spanish university graduates, followed biennially with mailed questionnaires. We identified 686 case-control pairs, matched for age and time in the cohort. Cases were women reporting difficulty getting pregnant. Controls did not consult due to difficulty conceiving and had at least one child during follow-up. After adjustment for potential confounders, we found no association between self-reported difficulty getting pregnant and the number of alcoholic beverages consumed per week, (Odds Ratio [OR] > 5 drinks/week *vs.* none = 1.04, 95% Confidence Interval [CI] = 0.72–1.51). No association between types of alcoholic beverage and difficulty conceiving (OR > 5 drinks of wine/week *vs.* none = 1.16, 95% CI = 0.72–1.88; OR > 5 drinks of beer/week *vs.* none = 1.06, 95% CI = 0.82–1.37; OR > 5 drinks of spirits/week *vs.* none = 1.24, 95% CI = 0.84–1.64) was observed. In conclusion, we found no association between alcohol intake and risk of consulting a physician due to difficulty conceiving. More studies are needed to clearly elucidate the effects of alcohol intake on women’s fertility. In the meantime, recommendations about alcohol intake to couples trying to conceive have to be given cautiously.

## 1. Introduction

It has been estimated that there are approximately 48.5 million infertile couples worldwide [1] with prevalence estimates ranging between 15% and 30% of couples [2,3]. Causes of infertility are varied and some of them, such as tubo-peritoneal factors and diminished ovarian reserve, are unmodifiable once identified. While potentially modifiable factors, such as smoking, obesity and underweight, are well-established risk factors for infertility, studies about other modifiable factors, such as diet, exercise, caffeine or alcohol intake, show inconsistent findings [4].

The role of alcohol on fertility, while extensively studied, remains unclear [4], especially on female fertility. In women, several studies observed a direct association between alcohol intake and the risk of infertility [5,6,7,8,9]. However, multiple studies have found no association between alcohol intake and female infertility [7,10,11,12,13] and a few studies even suggest a beneficial effect of alcohol on female fertility [14,15]. To shed further light on the relationship between alcohol intake and fertility, we investigated this association among female participants of the Seguimiento Universidad de Navarra (SUN) cohort.

## 2. Materials and Methods

### 2.1. Study Population

We conducted a case-control study nested in the SUN cohort. The SUN project started in 1999. It is a dynamic, ongoing and multipurpose prospective cohort of university graduates from all over Spain. Details of the project have been published elsewhere [16]. To date, more than 21,000 participants have been included in the cohort. Participants are followed up biennially with mailed questionnaires about diet, other lifestyle factors and health outcomes such as cardiovascular diseases or difficulty conceiving, among others. The retention rate in the cohort is approximately 92%.

The Institutional Review Board of the University of Navarra approved the study protocol. Voluntary completion of the first self-administrated questionnaire to enter into the SUN cohort was considered to imply informed consent.

For the present analysis, we selected 13,231 women recruited until December 2013. Among them, 8749 were aged 20–40 years and had a minimum follow-up of 2 years and a lag-time period of 9 months. After excluding women who were lost to follow-up (*n* = 782), those with extreme total energy intakes (<500 or >3500 Kcal/day) [17] (*n* = 691) and those who reported difficulty getting pregnant before study inception (prevalent cases) (*n* = 75), there were 7201 women available for the study. To avoid reverse causality bias, we decided to exclude women who had children when they entered into the cohort (*n* = 1914). On the other hand, to assure the fertility of the controls, we excluded those with no children during follow-up (*n* = 3583).

To identify the cases and the controls for this study, we used the question “Have you consulted a physician because of difficulty getting pregnant?” included in all biennial follow-up questionnaires. Incident cases were women answering affirmatively to this question during follow-up with no children when they entered into the cohort. Controls were women who answered negatively to the above-mentioned question, had no children when they entered into the cohort and had at least one child during follow-up. Cases and controls were matched for age and time at risk using an incidence density sampling approach. Accordingly, we had 686 case-control pairs available for the present analyses. Assuming an expected Odds Ratio (OR) of 1.6 [5], a 15% exposure probability among controls, a 0.2 correlation of exposure between pairs in the case-control set, and a type 1 error of 0.05 our statistical power was 86%.

### 2.2. Exposure Assessment

Dietary information was collected at baseline through a 136-item food-frequency questionnaire (FFQ) [18]. The FFQ included some questions on alcoholic beverages consumption (red wine, other wine, beer, and spirits). Each item had nine categories of consumption frequency (from never or seldom to six or more times per day). Spanish food composition tables were used to derive the nutrient composition of dietary intake [19]. This FFQ was previously validated for the Spanish population. The correlation coefficient between alcohol intake from the FFQ and from four food records was 0.90 [18]. Participants were classified in 4 groups according to their weekly alcohol intake: abstainers, those who drink 1 or less drinks/week, those who drink more than 1 and less than 5 drinks/week, and those who drink 5 or more drinks/week. The same categories were used to classify participants when exploring the association between consuming different alcoholic beverages (wine, beer and spirits) and difficulty conceiving.

### 2.3. Covariate Assessment

The baseline questionnaire inquired information about socio-demographic, anthropometric, and lifestyle variables, such as age, weight, height, physical activity, smoking status and parity. Weight (in kilograms) was divided by the square of height (in meters) to calculate the body mass index (BMI). Self-reported BMI was validated in a subsample of the cohort [20]. Physical activity was collected through a previously validated questionnaire [21]. Adherence to the Mediterranean dietary pattern was assessed with a score proposed by Trichopoulou *et al.* [22] but excluding alcohol intake in the calculation of this score to avoid overlapping with the main exposure. It scores positively if consumption of cereals, legumes, fish, fruit and vegetables, and the ratio monounsaturated/polyunsaturated fatty acids is beyond the median. It also scores positively if meat and meat products, and dairy products consumption is below the median. The sum of every point leads to a score that ranges from 0 (minimum adherence) to 8 (maximum adherence).

Questionnaires from the SUN project (in Spanish) are available online (http://www.unav.edu/departamento/preventiva/sun).

### 2.4. Statistical Analysis

We used conditional logistic regression models to evaluate the association between categories of weekly alcohol intake and the incidence of difficulty conceiving. We calculated odds ratios (OR) and their 95% confidence intervals (CI) using abstainers group as the reference category. We fitted a crude model, an age-adjusted model, and a multivariable-adjusted model that included the following potential confounders: BMI (<18.5, ≥18.5 to <25, ≥25 to <30, ≥30 kg/m^2^), smoking status (current smoker, former smoker, never smoker), leisure-time physical activity (METs-h/week), adherence to the traditional Mediterranean dietary pattern (MDP, 3 categories of adherence), use of vitamin supplements (yes/no). Analyses were also adjusted for total energy intake (Kcal/day). We also conducted tests for linear trend by introducing the number of drinks per week as a continuous variable in the model. All *p* values were two-tailed (*p* < 0.05 was considered statistically significant). 

Analyses were performed with the STATA statistical software package, version 12.2.

## 3. Results

**Figure 1:**
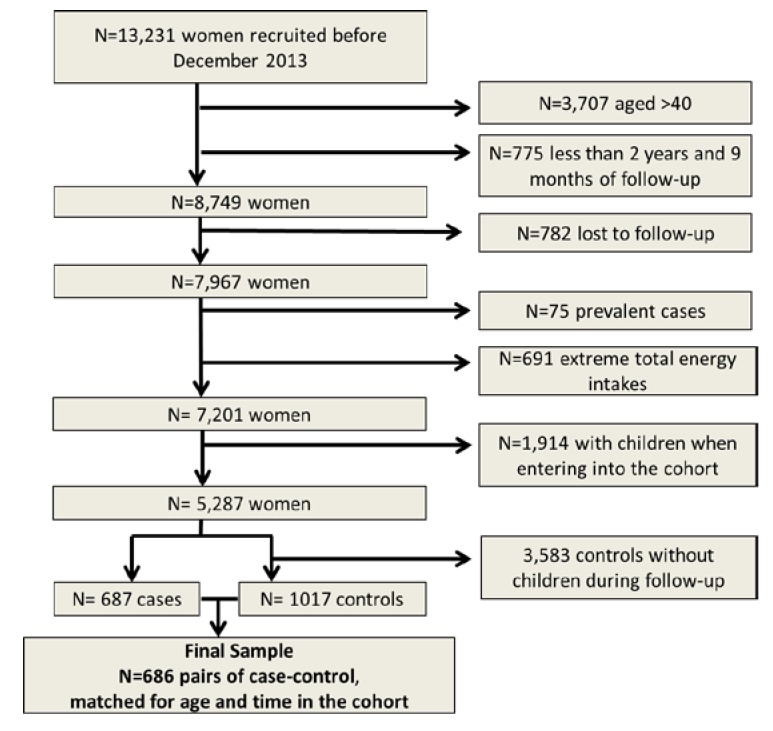
Flow chart of participants in the study

For the present analyses, we included 686 matched case-control pairs (Figure 1).

Baseline characteristics of the cases and the controls are shown in Table 1. There were more current smokers among cases than among controls. Obesity and use of vitamin supplements were more common in cases than in controls. Alcohol intake was low in both groups, especially consumption of spirits; mean alcohol intake was 0.35 g/day.

We did not find any association between the number of alcoholic beverages consumed per week and self-reported difficulty getting pregnant, even after adjustment for possible confounders such as BMI, smoking habit, physical activity, adherence to the Mediterranean diet, use of vitamin supplements and total energy intake (OR >5 drinks/week *vs.* none = 1.04, 95% CI = 0.72 − 1.51) (Table 2).

**Table 1:** Baseline characteristics of the cases (women with difficulty getting pregnant) and the controls from the Seguimiento Universidad de Navarra (SUN) study, matched for age and time in the cohort.

Baseline Characteristics *	Cases (*n* = 686)	Matched Controls (*n* = 686)
Age, years	29.3 (4.2)	29.3 (4.2)
Body mass index, kg/m^2^	21.41 (2.70)	21.26 (2.31)
Body mass index, *n* (%)		
<18.5	56 (8.2)	55 (8.1)
≥18.5 to <25	567 (82.6)	589 (85.8)
≥25 to <30	50 (7.3)	36 (5.2)
≥30	13 (1.9)	6 (0.9)
Smoking status, *n* (%)		
Never smokers	363 (53.9)	356 (53.2)
Past smokers	132(19.6)	204 (30.4)
Current smokers	179 (26.5)	110 (16.4)
Alcohol intake, g/day	0.35 (0.53)	0.36 (0.60)
Number of alcoholic, drinks/day		
Wine	0.16 (0.39)	0.17 (0.48)
Beer	0.11 (0.21)	0.11 (0.19)
Spirits	0.07 (0.14)	0.08 (0.15)
Physical activity, METs-h/week ^†^	18.7 (19.6)	19.1 (21.9)
Adherence to Mediterranean Diet ^#^	3.7 (1.6)	3.9 (1.5)
Use of vitamin supplements, *n* (%)	134 (19.5)	129 (18.8)
Total energy intake, Kcal/day	2210.6 (571.5)	2249.5 (546.9)

* Mean (standard deviation), unless otherwise stated; ^†^ METs: ratio of work metabolic rate to a standard resting metabolic rate; ^#^ Adherence to Mediterranean Diet (score from 0, minimum, to 8, maximum).

We also found no association when different alcoholic beverages were analyzed separately, even after adjustment for potential confounders: wine (OR >5 drinks/week *vs.* none = 1.16, 95% CI = 0.72 − 1.88), beers (OR >5 drinks/week *vs.* none = 1.06, 95% CI = 0.82−1.37) and spirits (OR >5 drinks/week *vs.* none = 1.24, 95% CI = 0.84−1.64). No significant linear trend was observed for any of the alcoholic beverages (Table 2).

**Table 2:** Odds ratio (95% CI) for presenting difficulty getting pregnant according to the consumption of alcoholic beverages/week.

	Consumption of Alcoholic Beverages (drinks/week)				
	Never or seldom	≤1/week	>1week to <5/week	≥5/week	*P* for trend
No. cases/controls	152/150	154/147	284/288	96/101	
Matched for age	1 (ref.)	1.04 (0.75–1.43)	0.97 (0.74–1.29)	0.94 (0.66–1.34)	0.71
Multivariable adjustment *	1 (ref.)	1.09 (0.78–1.53)	1.02 (0.76–1.37)	1.04 (0.72–1.51)	0.97
					
	Consumption of wine (1 unit = 1 glass of wine = 100 mL)				
	Never or seldom	≤1/week	>1week to <5/week	≥5/week	*P* for trend
No. cases/controls	312/317	177/164	156/166	41/39	
Matched for age	1 (ref.)	1.10 (0.84–1.42)	0.95 (0.72–1.25)	1.07 (0.67–1.70)	0.66
Multivariable adjustment *	1 (ref.)	1.10 (0.84–1.43)	0.97 (0.74–1.29)	1.16 (0.72–1.88)	0.84
					
	Consumption of beers (1 unit = 330 mL)				
	Never or seldom	≤1/week		>1/week	
No. cases/controls	326/334	152/140		208/212	
Matched for age	1 (ref.)	1.11 (0.84–1.45)		1.01 (0.78–1.29)	0.71
Multivariable adjustment *	1 (ref.)	1.15 (0.87–1.52)		1.06 (0.82–1.37)	0.98
				
	Consumption of spirits (1 unit = 50 mL)				
	Never or seldom	≤1/week		>1/week	
No. cases/controls	377/392	156/154		153/140	
Matched for age	1 (ref.)	1.05 (0.81–1.36)		1.13 (0.87–1.47)	0.86
Multivariable adjustment*	1 (ref.)	1.11 (0.85–1.45)		1.24 (0.84–1.64)	0.78

* Adjusted for BMI (4 categories), smoking status (3 categories), leisure-time physical activity (METs-h/week), use of vitamin supplements, adherence to the traditional Mediterranean diet and total energy intake. ref.: reference category.

## 4. Discussion

We prospectively evaluated the relationship between alcohol intake and female fertility, finding no associations between any level of alcohol intake and the risk of consulting a physician due to difficulty conceiving. Neither did we find associations between consumption of different types of alcoholic beverages and this outcome.

Several studies showed a deleterious effect of alcohol on female fertility [5,6,8,9,23], while others did not [7,10,11,13,14,24,25]. Inconsistences among studies are expected because several factors have to be taken into account to evaluate the effects of alcohol on fertility—for example, the amount and type of alcoholic beverage consumption, the potential confounders (age, parity, body mass index and lifestyle factors), the definition of the outcome (fecundability, waiting time to pregnancy, infertility due to a specific causes or medical consultation of infertility) and the design of the study and its possible biases [26]. The retrospective data collection may lead to misclassification and thus to biased results.

A case-control study among 4883 women found that moderate and high alcohol intake was associated with infertility due to ovulatory factor and endometriosis [23]. Regarding endometriosis, a frequent cause of infertility, a meta-analysis concluded that there was no association between any level of alcohol intake and endometriosis, although whether alcohol preceded endometriosis or exacerbated it was not clear in this study [24]. In our study, we did not analyze specific causes of infertility.

Another retrospective study evaluated moderate and high alcohol intake and waiting time to pregnancy in a Danish cohort of pregnant women, finding no association between them [14]. Neither did a Canadian study [13]. The European study on infertility and subfecundity, using a multicenter sample of randomly selected women aged 25–44 years, showed no association between alcohol intake and waiting time to pregnancy after adjusting for potential confounders [13].

Besides these, two prospective studies found that increasing alcohol intake was associated with a decrease of fecundability (probability of conception in a menstrual cycle), after adjusting for potential confounders (age, smoking, caffeine intake, *etc.*). Fecundability was reduced even with low alcohol intake [8,9]. It has been proposed that high and moderate intake of alcohol increase sex steroid hormones by stimulating androgen production in ovary and adrenal glands, increasing liver aromatase activity which converts androgens into estrogens or inhibiting liver enzymes to degrade estrogens. The increase in sex steroid hormones reduces FSH secretion and consequently inhibits ovulation [27,28,29]. However, our findings are not directly comparable to these studies, as our outcome was consulting a physician due to difficulty conceiving, not fecundability.

Another cohort study, conducted in a random sample of 7393 Swedish women, found that high alcohol intake compared to moderate consumers, but not low, was associated with increased risk of having medical examinations due to infertility [5], which was an outcome similar to ours. This inconsistency between Eggert’s study and ours could be explained by the fact that the Swedish study only adjusted for age but not for other potential confounders, such as smoking, BMI or parity, as we did. In fact, our results are consistent with other studies [10,11,13,14]. Chavarro *et al.* followed 18,555 fertile married women in the Nurses’ Health Study, measuring dietary factors and other potential confounders. Alcohol drinking was unrelated to ovulatory infertility [10]. This result is not surprising because alcohol may improve insulin sensitivity [30], which is related to a proper ovulatory function [31]. When comparing different alcoholic beverages, wine seemed to have greater beneficial effects on insulin resistance, mainly through its non-alcoholic components (the polyphenols) [30,32,33]. However, studies about wine and fertility are really scarce, especially prospective studies. In a retrospective study, Juhl *et al.* showed a protective effect of wine consumption on shorter waiting times of pregnancy compared with non-wine drinking or other alcoholic drinks [15]. Authors explained that wine drinking is generally associated with healthier lifestyles [34], but they did not control for those factors. In addition, they did not adjust for smoking. Therefore, their results could be biased. More studies evaluating the effect of wine consumption and the Mediterranean alcohol-drinking pattern on fertility are needed.

In our study, several limitations need to be considered. First, we evaluated the association between alcohol intake and difficulty getting pregnant, without specifying causes of infertility. We cannot rule out the possibility that associations with specific conditions in opposite directions could, when aggregated, result in a null finding. Second, we restricted the analysis to women with a university degree that had no children when entering the cohort. Therefore, results could not be generalizable to women with secondary infertility. In any case, the restriction that we applied by selecting only women with a high educational level can actually enhance the internal validity of our results because the high level of education and homogeneity of the cohort also with respect to similar access to health care reduce potential confounding related to socio-economic status. In addition, this characteristic increases the quality of the self-reported information provided by participants. Third, we are aware that characterization of infertility by one question is a crude way to select the targeted population and a non-differential misclassification of the outcome might have happened due to the self-reported nature of the information. But misreporting seems unlikely, as all women in the SUN cohort are highly educated. In fact, several conditions have been validated in this cohort (e.g., depression, metabolic syndrome, hypertension or physical activity), showing high accuracy [21,35,36,37]. Fourth, alcohol data were obtained from the baseline questionnaire of the SUN study and could have changed thorough the follow-up. In fact, considering that alcohol is a known teratogenic [38,39], many women trying to get pregnant may have avoided taking alcohol all together by the time willing to get pregnant or at least after ovulation. Since the information on alcohol consumption was based on a food-frequency questionnaire and these questionnaires collect the information on the long term, our work reflects the effect of the long-term alcohol consumption rather than a periconceptional short-term effect. Fifth, our questionnaires recall no information on contraceptive use. Thus, we could not take this factor into account as a potential confounder.

Despite these limitations, our study has several strengths. We selected cases and controls with no children when entering into the cohort and chose incident cases to avoid a possible reverse causation bias. It could be argued that nulliparous women might go out more frequently than those having any child, and consequently drink more alcohol, although the opposite could also be possible. In the former situation, infertility would be the “cause” of more alcohol intake instead of the outcome. Selecting nulliparous participants when entering into the cohort and choosing incident cases, as we did, helps to avoid this possible reverse causation bias. Other strengths are data collection and analysis. The high educational level of the participants provides good quality of information and high internal validity. It was also possible to adjust for possible confounders and to assure the fertility of the controls since we excluded those with no children during follow-up.

## 5. Conclusions

In conclusion, our results showed no association between alcohol intake and the risk of consulting a physician due to difficulty conceiving. Effects of alcohol on infertility deserve further investigation, taking into account that different effects are possible regarding the specific cause of infertility evaluated and the type of alcoholic beverage consumed. More studies are needed to clearly elucidate the effects of alcohol intake on women’s fertility. In the meanwhile, recommendations about alcohol intake to couples trying to conceive have to be given cautiously.
